# An Extensive Stanford Type A Aortic Dissection Involving Bilateral Carotid and Iliac Arteries

**DOI:** 10.1155/2013/607012

**Published:** 2013-01-14

**Authors:** E. W. Lee, N. Jourabchi, S. C. Sauk, D. Lanum

**Affiliations:** ^1^Department of Radiology, David Geffen School of Medicine, UCLA Health System, Los Angeles, CA 90095, USA; ^2^Department of Family Medicine, Arrowhead Regional Medical Center, Colton, CA 92324, USA; ^3^Mallinckrodt Institute of Radiology, Washington University, Campus Box 8131, 510 South Kingshighway Boulevard, St. Louis, MO 63110, USA

## Abstract

We present a rare case of continuous, extensive aortic dissection (AD) involving the bilateral common carotid arteries, the ascending, thoracic, and abdominal aorta, and bifurcation of the right common iliac artery. A 61-year-old man with history of chronic hypertension presented with a one-day history of chest pain, vertigo, left facial drooping, and left hemiparesis. Despite the presence of bilateral carotid bruits, doppler ultrasound of the neck was postponed, and the patient was treated with thrombolytic therapy for a presumed ischemic stroke. The patient's symptoms began to resolve within an hour of treatment, at which time treatment was withheld. Ultrasound performed the following day showed dissection of bilateral common carotid arteries, and CT angiography demonstrated extensive AD as described earlier. The patient subsequently underwent cardiovascular surgery and has been doing clinically well since then. AD has a myriad of manifestations depending on the involvement of aortic branches. Our paper illustrates the importance of having a high index of suspicion for AD when a patient presents with a picture of ischemic stroke, since overlapping signs and symptoms exist between AD and stroke. Differentiating between the two conditions is central to patient care as thrombolytic therapy can be helpful in stroke, but detrimental in AD.

## 1. Introduction

Aortic dissection is a life-threatening condition that affects 5–30 per 1,000,000 individuals each year [1]. Chronic hypertension is by far the most common risk factor for development of AD and accounts for 62–73% of patients with AD [1]. Other risk factors include diseases of the aorta (e.g., bicuspid valve, coarctation, aneurysm), connective tissue diseases (e.g., Marfan's, Ehrlo-Danlos), Turner syndrome, trauma, cocaine use (e.g., in normotensive patients), and previous cardiac surgery or catheterization [1]. It typically involves a tear in the intimal layer of the aorta and creation of a “false lumen” between the aortic intima and the media or adventitia. Subsequent propagation of the dissection can occur both proximally and distally, which may lead to severe intravascular volume loss, tissue ischemia, or cardiac tamponade if the ascending aorta is involved. One theory regarding development of AD suggests that the intimal tear, being the initial event, triggers subsequent dissection of the medial layer. Others, however, believe that an expanding hematoma in the medial layer (e.g., from rupture of the vasa vasorum) leads to the ensuing tear of the intima [2]. 

There exist two classifications of AD. For the purposes of our discussion, we will focus on the Stanford system. Stanford Type A is defined as any dissection that involves the ascending aorta; whereas type B is AD in the absence of ascending aorta involvement. This classification has important clinical implications; type A dissections are surgical emergencies while type B ADs are generally medically managed. The current literature suggests mortality rates of up to 2% per hour for type A dissections [3]. This is not surprising considering the fact that type A ADs, by definition, must involve the ascending aorta and are therefore more prone to catastrophic complications such as cardiac tamponade, myocardial infarction, and aortic regurgitation. 

AD classically presents with sharp or “tearing” chest pain that radiates to the back. However, other signs include blood pressure discrepancy in the upper extremities, Horner syndrome, and hoarseness. If the dissection extends into the carotid arteries, neurologic signs may be present as well. For instance, a patient may present with stroke-like symptoms, syncope, or decreased mental status. Consequences of type B ADs, on the other hand, are mostly related to ischemia of the organs and tissues distal to the tear.

We present here a case of an extensive type A aortic dissection from the ascending aorta to the right common iliac arteries with additional propagation to the common carotid arteries bilaterally. 

## 2. Case

The patient is a 61-year-old Hispanic male who presented to the emergency department (ED) with a one-day history of vague chest pain which began overnight, vertigo, left facial drooping, left hemiparesis, and a single episode of syncope. His past medical history was significant for mild hypertension and atherosclerosis. On physical exam, patient was noted to have bilateral carotid bruit, a III/VI diastolic murmur at the left sternal border. Otherwise, pulses were present and undiminished, and vital signs were stable and within normal ranges on presentation. Chest X-ray obtained in the ED showed aortic calcifications consistent with history of atherosclerosis without significant mediastinal widening. Patient at this point also had an elevated d-dimer of 18,600. Given his focal neurologic symptoms (e.g., left facial drooping and left hemiparesis), bilateral carotid bruit, and no evidence of intracranial bleed on head CT, the patient was empirically started on thrombolytic therapy (i.e., tissue plasminogen activator, tPA) for presumed stroke. Per ED documentation, patient's symptoms started to resolve within an hour upon arrival, and tPA was discontinued accordingly.

**Figure 1:**
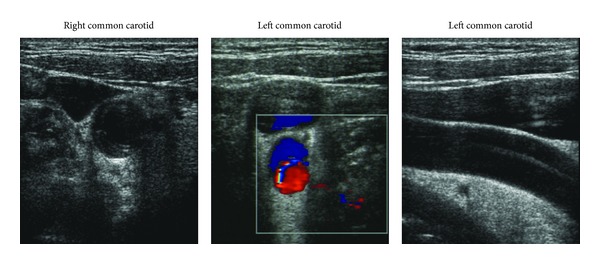
Doppler ultrasounds showing bilateral common carotid artery dissection.

**Figure 2:**
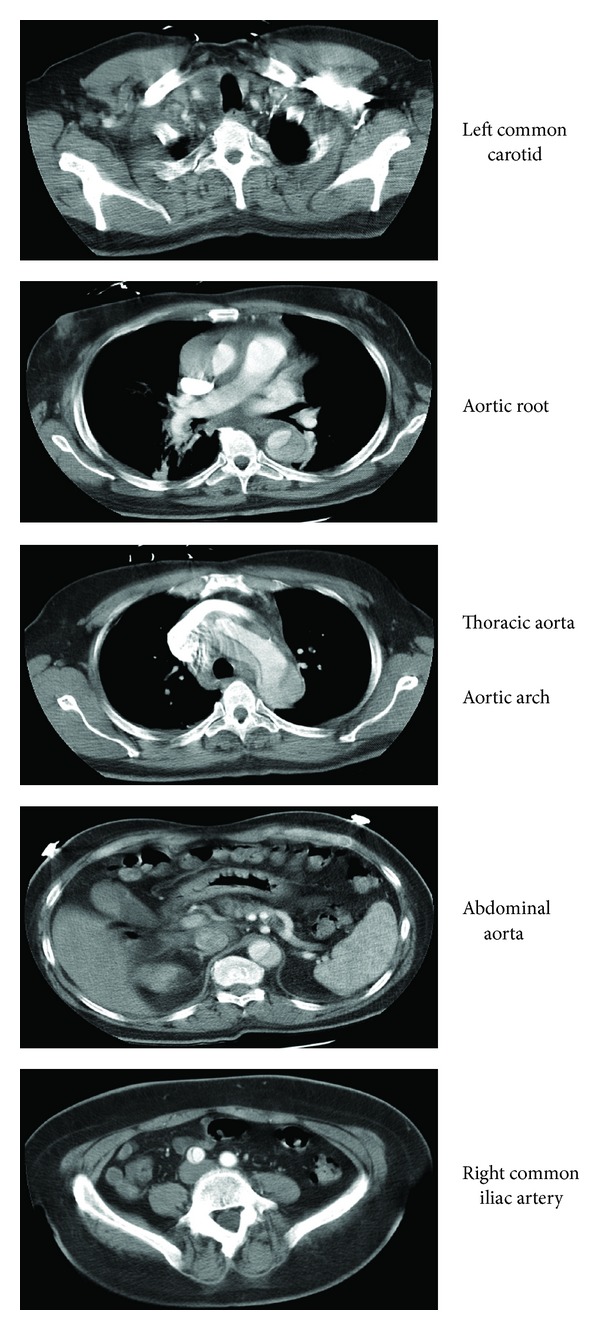
CT angiogram of the chest, abdomen, and pelvis revealed a complete aortic dissection from the aortic ascending upward through the common carotid arteries and downward through the thoracic aorta and abdominal aorta with a final intimal flap in the right common iliac bifurcation.

Further workup demonstrated dissections of the common carotid arteries bilaterally on Doppler ultrasound (Figure 1). CT angiogram of the chest, abdomen, and pelvis revealed a complete aortic dissection from the aortic ascending upward through the common carotid arteries and downward through the thoracic aorta and abdominal aorta with a final intimal flap in the right common iliac bifurcation (Figure 2). Given the extensive involvement of the aortic dissection, patient was transferred within an hour of completion of CT scan to a nearby medical facility with a cardiothoracic surgery service.

At surgery, patient was noted to have moderate hemopericardium. Crossclamping of the ascending aorta was achieved after establishment of a full cardiopulmonary bypass connecting the right atrium to the right femoral artery. Next, temporary cardiac arrest under hypothermia was maintained with a cardioplegic solution. An aortic tear 5 cm above the sinotubular junction was initially seen, and the aorta was transected. Obliteration of the false lumen created by the tear was done by sealing of the intimal and medial layers with BioGlue (CryoLife, Kennesaw, Georgia). The proximal and distal ends of the aorta were then reconnected with a number 28 Vascutek graft. A second tear was surgically repaired proximal to the brachiocephalic trunk. Total crossclamp time was 113 minutes, and total cardiopulmonary bypass time was 134 minutes. Circulatory arrest lasted for 7 minutes. Patient tolerated the procedure well. He was intubated and transferred to the intensive care unit postoperatively. On post-op day 5, patient was deemed hemodynamically stable and transferred to cardiac critical care unit for further observation. Patient was finally discharged home on antihypertensive medications and followed up post-operatively with vascular surgery service. A repeat Doppler was done one week post-op which showed persistent dissections of both common carotid arteries; however, there was no evidence of stenosis, and both vertebral arteries remained patent. Patient also continued to be seen by outpatient cardiology on a regular basis and has done clinically well since his surgery, as evidenced by well-controlled blood pressures. 

## 3. Discussion

Spontaneous aortic dissection involving both common carotid arteries, the entire length of the aorta, and the right common iliac artery is rare in the existing literature. Beside a history of chronic hypertension, our patient had no other significant predisposing factors for AD, such as a history of trauma, prior cardiac surgical intervention, or known connective tissue disease. A few case reports of spontaneous dissection of bilateral common carotid arteries do exist; however, none to the same extent of aortic involvement as our case exist. Yeh et al. reported a case of a 56-year-old woman with vague chest pain and focal neurologic deficits whose decreased mental status on initial presentation made it difficult for a thorough medical history to be obtained. Diagnosis relied on CT angiography which delineated a type A AD involving both CCAs [4]. Similarly, Demiryoguran et al. [5] reported a 63-year-old female with a predominant symptom of vertigo who was also found to have AD involving the ascending aorta, aortic arch, and bilateral common carotid arteries. Both cases prove that the timely use of Doppler ultrasound after bilateral carotid bruits are heard on physical exam is an important next step in diagnosis. Doppler ultrasound findings in these cases as well as ours revealed bilateral common carotid artery dissection, which prompted confirmation with highly diagnostic imaging studies such as CT and/or CT angiography. Our patient had several significant findings on exam, including bilateral carotid bruits and a diastolic murmur suggestive of aortic regurgitation (heard in 40–50% of patients with proximal AD [3]). Despite these clinical clues, a Doppler ultrasound was not done immediately in the ED but was instead postponed until the next day. This delayed both the acquisition of CT angiography and transport of the patient to a nearby medical facility with vascular surgery capabilities. Luckily, the patient tolerated the surgery well and has been doing well since the surgery.

Our paper also demonstrates the importance of a complete workup of all major aortic branches after aortic dissection at a specific location such as the CCA has been confirmed. Unlike the aforementioned cases of bilateral CCA dissection, our patient had additional dissection of the entire length of the aorta down to the right common iliac bifurcation. Even in the absence of trauma, it is equally imperative that all branches of the aorta be scanned for ancillary involvement in order to better delineate the extent of the dissection and plan for medical or surgical management accordingly.

Our patient's initial presentation of nonspecific chest pain, vertigo, left facial drooping, left hemiparesis, and a previous episode of syncope seems to defy the classical picture of AD (i.e., tearing or ripping chest pain that radiates to the back). As mentioned previously, depending on the extent of the dissection and any involvement of aortic branches, patients with AD may present with a wide range of symptoms and signs that may be common in other conditions as well, such as ischemic stroke, and myocardial infarction. Specifically, neurologic manifestations have been reported to occur in 18–30% of patients with AD with stroke symptoms being the most common, occurring in 5–10% of patients [1]. Decreased cerebral perfusion from complications of ascending aortic dissection itself, such as cardiac tamponade or stroke, can result in altered mental status or syncope [6]. Nallamothu et al. in 2002 demonstrated that AD patients who presented with syncope fared worse and had higher rates of in-house mortality than their conscious counterparts [6].

In the case of our patient, a presumptive diagnosis of ischemic stroke was made initially given the overlapping neurologic symptoms between AD and stroke and the patient was incorrectly started on tPA. This would have been a catastrophe had the physicians not withheld the tPA in time. There have been several cases reported of spontaneous aortic dissection which presented with ischemic stroke-like signs and symptoms. Grupper et al. in 2007 reported a 77-year-old patient who, similar to our patient, initially presented with predominantly neurologic manifestations, including decreased mental status, left hemiplegia, positive Babinski's on the left, and gaze deviation to the right. Patient had otherwise normal pulses distally and 2+ reflexes [7]. Chest X-ray and a CT of the head were negative for mediastinal widening and intracranial bleed, respectively. Fortunately in this case, patient's symptoms resolved spontaneously before receiving his first dose of tPA. A final diagnostic CT of the chest and abdomen revealed aortic dissection of the thoracic and abdominal aorta, left renal, superior mesenteric artery and the right external carotid artery. 

One study suggests that roughly 38% of ADs are missed on initial presentation [1]. Considering these staggering statistics, a thorough evaluation of AD will rely not only on a complete history and physical examination, but also on various diagnostic imaging modalities such as CXR, CT, and transesophageal echocardiogram (TEE). Furthermore, a high degree of clinical suspicion for AD should always be exercised whenever a patient presents with “classical” symptoms of ischemic stroke, in order to avoid the devastating consequences of initiating thrombolytic therapy in patients without an ischemic stroke.
